# Patterns of Recombination in HIV-1M Are Influenced by Selection Disfavouring the Survival of Recombinants with Disrupted Genomic RNA and Protein Structures

**DOI:** 10.1371/journal.pone.0100400

**Published:** 2014-06-17

**Authors:** Michael Golden, Brejnev M. Muhire, Yves Semegni, Darren P. Martin

**Affiliations:** 1 Department of Statistics, University of Oxford, Oxford, United Kingdom; 2 Institute of Infectious Diseases and Molecular Medicine, Computational Biology Group, University of Cape Town, Cape Town, South Africa; 3 Department of Mathematics and Physics, Cape Peninsula University of Technology, Cape Town, South Africa; North Carolina State University, United States of America

## Abstract

Genetic recombination is a major contributor to the ongoing diversification of HIV. It is clearly apparent that across the HIV-genome there are defined recombination hot and cold spots which tend to co-localise both with genomic secondary structures and with either inter-gene boundaries or intra-gene domain boundaries. There is also good evidence that most recombination breakpoints that are detectable within the genes of natural HIV recombinants are likely to be minimally disruptive of intra-protein amino acid contacts and that these breakpoints should therefore have little impact on protein folding. Here we further investigate the impact on patterns of genetic recombination in HIV of selection favouring the maintenance of functional RNA and protein structures. We confirm that chimaeric Gag p24, reverse transcriptase, integrase, gp120 and Nef proteins that are expressed by natural HIV-1 recombinants have significantly lower degrees of predicted folding disruption than randomly generated recombinants. Similarly, we use a novel single-stranded RNA folding disruption test to show that there is significant, albeit weak, evidence that natural HIV recombinants tend to have genomic secondary structures that more closely resemble parental structures than do randomly generated recombinants. These results are consistent with the hypothesis that natural selection has acted both in the short term to purge recombinants with disrupted RNA and protein folds, and in the longer term to modify the genome architecture of HIV to ensure that recombination prone sites correspond with those where recombination will be minimally deleterious.

## Introduction

Recombination is a process involving the movement of genetic information within or between DNA/RNA molecules. Homologous recombination, where the fragment of transferred genetic information replaces a homologous fragment within its destination DNA/RNA molecule, is important from an evolutionary perspective because within individual genomes it can both remove harmful mutations and facilitate the accumulation of beneficial mutations. By creating novel combinations of nucleotide polymorphisms homologous recombination can also enable far wider exploration of a sequence space than is achievable by mutation alone [1], [2].

Homologous recombination, hereafter referred to simply as recombination, features prominently in the evolution of many viruses. In these organisms recombination does not necessarily involve the breakage and re-ligation of DNA/RNA molecules. In retroviruses such as HIV, for example, it predominantly occurs when RNA copies of the viral genome are being reverse transcribed into DNA by the viral enzyme, reverse transcriptase [3]–[5]. Every HIV virion contains two complete genomes (i.e. HIV is a diploid virus) and the reverse transcriptase will generally switch between these an average of approximately two to four times per replication cycle [6], [7]. If the two co-packaged HIV genomes are genetically different then such template switching could yield a detectably recombinant genome.

Although the capacity to recombine can provide viral species with a number of evolutionary benefits, many of the individual recombination events that occur between any particular pair of viruses will be deleterious; especially if they occur between distantly related genomes [8]–[10]. By bringing together divergent genome fragments that have largely independent evolutionary histories, recombination can potentially cause disruptions in coevolved intra-genome interaction networks [11], [12]. Examples of intra-genome interactions include base-pairing interactions in RNA structures, sequence specific protein-DNA interactions, interactions between proteins (inter-protein interactions) and interactions between amino acids within three-dimensional protein folds (intra-protein interactions). Natural selection should disfavour the survival of recombinant genomes in which such interactions are disrupted and it is therefore expected that patterns of recombination evident within circulating viruses might display evidence of such selection.

It has been demonstrated in *in vitro* protein evolution experiments that the most viable of the chimaeric proteins that are expressed from recombinant genes tend to have lower degrees of predicted folding disruption relative to wild-type proteins than do randomly generated chimaeras [11], [12]. Importantly, similar observations have been made when extending this approach to the analysis of chimaeric virus proteins (including those of HIV) that both occur naturally [13]–[15] and emerge during evolution experiments [10], [16], [17]. An obvious explanation of these tendencies is that recombinants expressing chimaeric proteins in which certain necessary intra-protein amino acid interactions are maintained will have a higher likelihood of replicating and surviving, whereas those that don't will be purged by selective processes.

Besides potentially disrupting intra-protein amino acid interactions, it is similarly possible that whenever biologically functional nucleic acid secondary structures are present within virus genomes, recombination could disrupt nucleotide-nucleotide interactions within these. When in their single-stranded RNA configuration, HIV genomes have a high degree of secondary structure, much of which is potentially biologically functional [18]. It is expected that recombinants in which biologically functional secondary structures are undisrupted should be more viable than those in which they are disrupted and, therefore, that natural recombinant genomes might display lower degrees of predicted secondary structural disruption than is expected if the maintenance of secondary structures had no evolutionary significance. While evidence of this has been observed amongst recombinant virus genomes arising during *in vitro* recombination experiments [17], it remains to be discovered whether such selection might have a detectable impact on patterns of virus recombination that arise under natural conditions.

Here we test whether the distinctive recombination patterns evident within naturally occurring HIV genomes [14], [19]–[21] display signs of selection disfavouring the survival of recombinants with either recombinationally disrupted intra-protein interactions or recombinationally disrupted RNA secondary structures. While we confirm previous findings that there is strong evidence in many HIV genes of natural selection disfavouring the survival of recombinants expressing misfolded chimaeric proteins [14], [15] we additionally find, for the first time in viruses sampled from nature, evidence that natural selection also disfavours the survival of genomes with recombinationally disrupted genomic secondary structures.

## Methods

### Dataset construction

A set of aligned patient derived HIV-1 group M sequences was obtained that had been previously analysed in great detail to characterise inter- and intra- subtype recombination events (i.e. to estimate recombination breakpoint locations and identify both the recombinant sequences and sequences resembling their parents; [14]). This alignment was originally retrieved from the Los Alamos National Laboratory (LANL) HIV Sequence Database (http://hiv-web.lanl.gov/) and contained a maximum of three reference sequences for each HIV-1M subtype, up to two sequences for each of 53 recognised HIV-1M circulating recombinant forms and 197 apparently unique recombinants. The alignment was trimmed to include only the protein coding sequences of the 274 genomes. The 434 recombination events detected within the sequences of this dataset were all manually checked with a range of breakpoint localisation and recombinant sequence identification tools available within the program RDP4 [22] to yield a fairly accurate list of recombination events (this dataset is available for download from http://computingforbiology.org/patterns-of-recombination-in-hiv both as an alignment in FASTA format, and a RDP4 readable file containing information on all of the analysed recombination events). A recombination event is considered here to be a recombinant sequence, a set of two breakpoints within the recombinant sequence and the identities of sequences in the dataset that most closely resemble the parental sequences that recombined in order to form the recombinant sequence. For any particular recombination event we differentiate between the parental viruses by referring to the sequence in the dataset that most closely resembles the sequence that provided the biggest fraction of the recombinant's genome as the “major” parent and the sequence resembling that which provided the smaller fraction as the “minor” parent.

PDB files containing the three-dimensional atomic coordinates of different protein crystal structures were downloaded from the Protein Data Bank (PDB, [23]). The PDB files corresponding to protein structures used in the recombination protein-fold disruption test are presented in Table 1.

**Table 1:** List of PDB structure files used in the chimaeric protein-fold disruption tests.

Protein name	PDB ID	Citation
p24	3H4E	[33]
gp41	1SZT	[34]
gp120	3JWD	[35]
Integrase	1K6Y	[36]
Nef	4D8D	Unpublished
Protease	3TKG	[37]
RNase	3LP3	[38]
RT	3KLF	[39]

### Simulating recombinants

**Figure 1:**
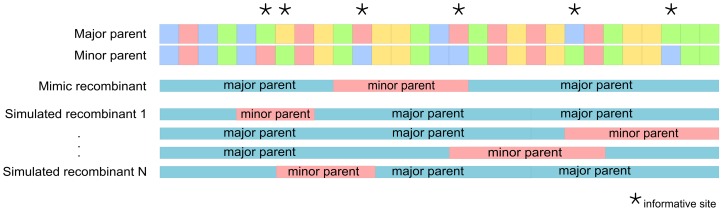
Diagrammatic representation of how simulated recombinants were generated. For a particular recombination event specifying a major parent, a minor parent, and a pair of recombination breakpoint locations delineating a fragment of sequence derived from the minor parent (containing in this particular case two nucleotides that vary between the major and minor parents), an *in silico* mimic of the real recombinant sequence is created using the minor and the major parent sequences. Following that, a set of N simulated recombinants is generated in a similar way to the mimic recombinant, but using random starting and ending positions, whilst maintaining the same number of either variable nucleotides (for the RNA folding tests) or non-synonymous codon sites (for the protein folding tests) between the randomized breakpoint sites as occur in the mimic recombinant. In this example the mimic and simulated recombinants all have two such “informative” sites between the 3′ and 5′ breakpoints that are not identical between the parental sequences.

The protein folding and secondary structure disruption tests performed here both relied on a permutation test involving the generation of sets of simulated recombinants with precisely the same genetic distances to the parental viruses but with breakpoints in random genome locations. Genetic distances were computed as the number of nucleotide differences between a pair of sequences (treating gap characters inserted during alignment as a fifth nucleotide state). Given the breakpoint positions and parental sequences associated with a particular recombination event, an *in silico* generated recombinant sequence with breakpoint positions corresponding to those of a real recombinant was produced from the minor and major parental sequences. *In silico* generated recombinants of this type were called “mimic” or M-recombinants in that although they resembled actual recombinants at the moment when these were generated, they were not expected to be identical to these actual recombinants. This is because the parental sequences, rather than being the actual parental sequences of the recombinant (it is extremely unlikely that these would ever be sampled), were simply those identified in our dataset as most closely resembling the actual parents. For each detected recombination event we refer hereafter to the 5′ breakpoint in its corresponding M-recombinant as the “start position”, the 3′ breakpoint as the “end position”, and the number of sites differing between the major and minor parents between these two positions as the “event-length”. For each of the M-recombinants, multiple simulated recombinant sequences, called S-recombinants, were then generated from the same major and minor parental sequences and with the same event-length but with randomly selected start positions (such that the end positions were simply determined by the event-length). Start positions that resulted in end positions falling beyond either the end of the gene of interest (for the protein folding disruption tests) or the end of the genome alignment (for the RNA folding disruption tests) were excluded. Keeping the event-length constant between the M- and S-recombinants ensured that these all had either exactly the same number of polymorphic translated amino acid sites (for the protein folding test) or exactly the same genetic distance (for the RNA folding tests) from both the major and minor parental sequences – something that was crucial for our permutation-based tests of recombination-induced protein and nucleic acid structural disruption. See Figure 1 for a diagrammatic representation of this procedure.

### Protein folding disruption tests

This test is based on that presented by Lefeuvre *et. al.* 2007 [13], and was performed using RDP 4.20 [22]. The test compares M- and S-recombinant sequences to their minor and major parental sequences in order to evaluate whether degrees of protein fold disruption, estimated using the SCHEMA method [11], are significantly lower in the M-recombinants than in the S-recombinants. Specifically, this test evaluates whether recombinant sequences display a degree of protein fold disruption that is lower than can be accounted for by chance under random recombination in the absence of natural selection. As input, the SCHEMA method takes a PDB protein structure file and parental amino acid sequences that are homologous to those within the PDB file. Assuming identical folding of the major and minor parental amino acid sequences to that represented within the PDB file, the SCHEMA method identifies pairs of potentially interacting amino acid residues as any pair of amino acids with any atoms that are within 4.5 ångströms of one another. A 4.5 ångström distance between two amino acid residues will correspond to approximately 5 to 8 atomic interactions between the residues [11]. The amino acid contact map thus generated is then used to determine the degree of expected fold disruption within a chimaeric protein that is expressed from a recombinant gene. At all pairs of potentially interacting amino acid sites where the parental amino acid sequences differ from one another (i.e. polymorphic sites), SCHEMA counts the number of these where one amino acid of the potential interacting pair comes from the minor parent and the other comes from the major parent. This number, called the E-score, has previously been shown to be highly correlated with degrees of protein fold disruption [11].

In order to test whether recombinant protein sequences avoid protein fold disruption more than can be accounted for by chance, random recombination events were simulated in the manner described above – each of the real recombination events was shifted up or down along the sequence by a random amount, whilst maintaining the same number of polymorphic non-synonymous codon sites (i.e. sites within homologous codons that encode different amino acids) as in the real recombinant. E-scores were calculated for each of the M-recombinants and their corresponding sets of S-recombinant proteins (1000 for each M-recombinant) and a permutation test was performed which counted the fraction of times that the sum of E-scores for the set of real M-recombinant proteins were less (i.e. the M-recombinants collectively had lower over-all degrees of estimated disruption) than the sum of E-scores for each of 100,000 sets of S-recombinant proteins. This fraction corresponds to the p-value that the real recombination events that are collectively represented amongst all the M-recombinants are not collectively less disruptive of protein folding than are those represented amongst their corresponding sets of S-recombinants: For a particular gene a p-value <0.05 therefore suggests >95% confidence that recombination events detectable under natural conditions within that gene are less disruptive of protein folding than would be expected in the absence of either (1) selection disfavoring the survival of viruses that express chimaeric proteins with disrupted folds or (2) mechanistic factors that cause recombination events to occur most frequently at genomic sites where they will have minimal impact on protein folding.

### Nucleic acid fold disruption tests

Tests for recombination-induced RNA secondary structure disruption that we performed were similar to those used to test for protein tertiary structure disruption. For each detected recombination event, one M- and ten S-recombinant sequences were generated and each of these sequences was computationally folded along with the major and minor parental sequences using an optimised version of the UNAfold program [24], [25]. In total there were 434 M-recombinants generated along with 4340 simulated recombinants. Differences in the folds between the parental sequences and those of their corresponding M- and S-recombinant sequences were quantified to determine whether the secondary structures of the M-recombinant sequences were collectively significantly less different to those of the parental sequences than were those of the S-recombinants. We used a variety of different tests to quantify the differences between the M- or S-recombinants and the parental sequences:

1. The aberrant base-pair test: the number of predicted base-paired nucleotides that were present in a M/S-recombinant, but were not present in either of the parental sequences.
2. The base-pair disruption test: the number of predicted base-paired nucleotides that were present in both parental sequences, but were not present in the M/S recombinant.
3. The minor parent base-pair disruption test: the number of predicted base-paired nucleotides that were present in the minor parent, but were not present in the M/S recombinant.
4. The major parent base-pair disruption test: the number of predicted base-paired nucleotides that were present in the major parent, but were not present in the M/S recombinant.

For each of these metrics we obtained (1) a list of disruption scores for the M-recombinants and (2) a ten times longer list of disruption scores for the S-recombinants. These lists were compared using a one-tailed Wilcoxon-rank sum test to determine whether the disruption scores of the M-recombinants were significantly lower than those of the S-recombinants. In these tests low disruption scores for the M-recombinants coupled with an associated p-value <0.05 would indicate with >95% confidence that recombination events detectable within HIV genomes are less disruptive of RNA folding than would be expected in the absence of either (1) selection disfavoring the survival of viruses in which recombination has disrupted RNA folding or (2) mechanistic factors that cause recombination events to occur most frequently at genomic sites where they will have a reduced impact on RNA folding.

## Results and Discussion

### Confirmation that selection favouring the avoidance of protein fold disruption clearly influences HIV-1 recombination patterns

Recombination that occurs between divergent genome fragments having largely independent evolutionary histories can potentially disrupt coevolved intra-genome interactions such as those occurring between amino acids within three-dimensional protein folds (i.e. intra-protein interactions). Here chimaeric proteins resembling those expressed by actual viruses were computationally tested to determine whether they displayed lower degrees of predicted folding disruption than those of randomly generated protein chimaeras. Actual recombinants sampled from nature would be expected to display less disruption of intra-protein interactions than simulated recombinants either if natural selection disfavoured the survival of recombinants expressing misfolded proteins, or recombination breakpoints tended to coincidentally occur most frequently at sites where they would have minimal impact on protein folding.

**Figure 2:**
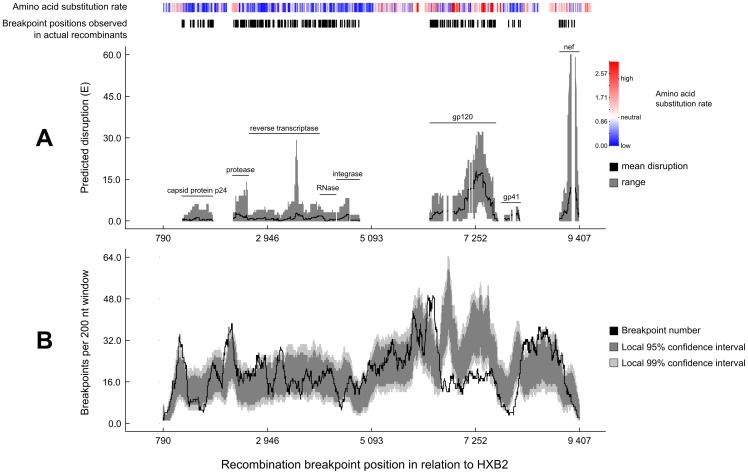
The predicted sensitivity of HIV-1M proteins to recombinational disruption. (**A**) Depicted are the means (black lines) and ranges (gray backgrounds) of predicted degrees of recombination-induced folding disruption in various HIV-1 proteins (those for which suitable atomic resolution three dimensional structures are available). The white areas interspersed between the gray areas are positions where there was no protein structure data available or where there were extra amino acids inserted into the alignment that were not present in the protein structure used. For all genome regions that had associated protein structure data, all conceivable single breakpoint recombinants were simulated using parental sequences that resembled as closely as possible the parental sequences of actual recombinant viruses with single detectable recombination breakpoints in these genome regions. Amino acid substitution rates and breakpoint positions occurring in these actual HIV-1 recombinants are displayed at the top of the figure. (**B**) Recombination breakpoint density plot illustrating breakpoint positions detected across 434 detectable HIV-1M recombination events (After [14]). Light and dark grey areas respectively indicate the 95% and 99% confidence intervals of breakpoint numbers that would have been detectable in different genome locations under random recombination. The grey areas undulate with degrees of sequence conservation because recombination events are more easily detectable in genome regions that are genetically diverse. Note firstly that the peaks of the plots in **A** indicate recombination breakpoint positions that are predicted to have the greatest disruptive effects on protein folding, and secondly that in actual recombinant HIV-1M genomes sampled from nature these “disruptive breakpoint positions” tend to correspond in plot **B** with regions of low recombination breakpoint densities.

Figure 2A illustrates degrees of intra-protein amino-acid – amino-acid interaction disruption (where higher E-values equate with greater disruption) that are predicted to occur within various HIV-1 proteins between HIV variants that have previously been observed to recombine (Figure 2B). The average degrees of predicted recombination-induced folding disruption in the gp120 and Nef proteins are appreciably higher than those predicted in the other HIV-1 proteins for which extensive high resolution structural data is available. Consistent with the notion that these proteins may be particularly sensitive to recombination-induced folding disruption is the fact that, in actual HIV-1M recombinants, breakpoints only very rarely occur at sites where they are anticipated to have a maximally disruptive effect on the folding of these proteins. A plausible explanation for gp120 and Nef being particularly sensitive to recombination-induced folding disruption relative to the other HIV-1M proteins examined here, is that they are less conserved than these other proteins (see amino acid substitution rate plot in Figure 2A) and recombinant versions of gp120 and Nef will therefore tend to have many more potentially disruptive amino acid combinations.

In order to more rigorously test whether the chimaeric proteins that are expressed by HIV-1M recombinants tend to display lower degrees of protein folding disruption than can be accounted for under random recombination in the absence of selection against misfolded protein chimaeras, individual HIV-1M proteins were analysed using a previously described permutation-based “avoidance of protein folding disruption” test [13].

Similar to the findings of a recent study using an alternative approach to that described here [15], we found that in five out of the eight analysed proteins, intra-protein amino acid interactions in chimaeric proteins expressed by natural HIV-1M recombinants are inferred to have been significantly less disrupted than could be accounted for by chance (Table 2). The main difference between our result and that of [15] is that we did not detect any evidence of avoidance of protein folding disruption in the protease protein.

**Table 2:** Degrees of protein fold disruption in natural and simulated HIV-1 recombinants.

Protein	Number of breakpoints	Mean E-score of M-recombinants	Mean E-score of S-recombinants	p-value^1^	p-values determined by Woo et al., (2014)	
					CC model^2^	MI model^3^
**p24**	18	0.059	0.478	0.0106	0.002	<0.001
**Protease**	16	1.875	1.971	0.5042	0.031	<0.001
**RT**	67	0.321	1.216	<0.0001	<0.001	<0.001
**RNase**	22	0.818	1.044	0.3260	ND^4^	ND
**Integrase**	21	0.211	0.868	0.0189	0.063	0.125
**gp120**	51	5.465	9.365	<0.0001	0.015	<0.001
**gp41**	5	1.000	1.954	0.1618	ND	ND
**Nef**	14	2.091	5.502	0.0185	ND	ND

1 The p-value is the probability that mimic recombination breakpoints do not tend to avoid disrupting protein folding to a greater degree than S-recombinants.

2 Covarying contact model of coevolution. Amino acids within van der Waals contact in the 3D structure were considered to be potentially covarying. The p-value is determined from a comparison of observed numbers of coevolving residues that are segregated by recombination with numbers predicted under random recombination.

3 Mutual information model of coevolution. Amino acids in contact in the 3D structure with associated mutual information values >0.25 were considered to be potentially covarying. P-values were determined as in ^2^.

4 Not determined.

Although three of the eight proteins analysed here displayed no detectable signal of lower than expected recombination-induced fold disruption (Protease, RNase and gp41), in at least one case (gp41), this may simply be due to low numbers of recombination breakpoints having been detected within the gene encoding this protein: a fact which reduces our ability to detect a signal in this protein. In this regard it is noteworthy that for all three of these proteins, the mean estimated fold-disruption in the M-recombinants was consistently lower than that of the S-recombinants (compare the E-scores in Table 2) which suggests that given either more data or more powerful fold disruption tests, it might be possible to demonstrate that these proteins too display lower than expected degrees of recombination-induced fold disruption.

There are two non-mutually exclusive potential explanations why breakpoints in natural recombinants might occur at genomic sites where they minimise protein fold disruption. The most obvious of these explanations is that the expression of a misfolded chimaeric protein is expected to have a negative impact on a virus' fitness such that recombination patterns observable amongst viruses sampled from nature will largely reflect the consequences of selection disfavouring the survival of viruses that express such proteins. The less obvious, but not less plausible, explanation is that HIV-1M genomes are mechanistically predisposed to accumulate recombination breakpoints at locations that minimise the chances of low-viability recombinants arising. Specifically, RNA secondary structures within HIV-1M genomes have a strong influence both on where recombination breakpoints are likely to occur [26] and on how proteins are likely to fold [18]. It is therefore been proposed that RNA structures occurring both at the junctions of different genes and at sites encoding the boundaries between discrete protein domains, may “direct” recombination to preferentially occur at locations in genes where it will have a minimal impact on protein folding [26].

### Avoidance of RNA folding disruption has a detectable influence on HIV-1 recombination patterns

Besides influencing where recombination events are most likely to occur within HIV genomes [26]–[29], RNA structures could also influence which recombinants are likely to survive. If, for example, a recombination breakpoint occurs within the sequence of a biologically functional hairpin structure it is possible that nucleotide differences between the parental genomes will cause destabilisation of the structure, and, consequently, a reduction of the resulting recombinant's fitness. A set of tests similar to that used to investigate recombination-induced protein folding disruption was devised to test whether recombination-induced RNA folding disruption has had a detectable influence on recombination breakpoint distributions that are detectable in natural HIV-1M recombinants.

Whereas the first of these tests measured the number of aberrant base-pairs in the recombinant secondary structures (an aberrant base-pair being a base-pair which is present in a M-/S-recombinant's secondary structure, but is not present in either of its parents' secondary structures), tests 2 to 4 examined whether individual base-pairs within the minor and major parent secondary structures were maintained in the secondary structures of M-/S- recombinants.

The results of test 1 indicate that M-recombinants tend to have significantly fewer aberrant base-pairs than S-recombinants (medians of 259 and 308 aberrant base-pairs, respectively; p-value  = 0.019). The results of test 2 suggest that the number of disrupted base-pairings (base-pairings predicted to be present in both of the parental sequence secondary structures, but not in the recombinant secondary structure) in the M-recombinants was significantly lower than those in the S-recombinants (medians of 52 and 70 disrupted base-pairs, respectively; p-value  = 0.005). These two tests collectively imply that, relative to parental sequences, the M-recombinants have significantly better preserved base-pairing configurations than do the S-recombinants.

Tests 3 and 4 relaxed the criterion in test 2 that considered only base-pairs that were present in both the minor and major parental sequence secondary structures. Specifically, test 3 counted the number of disrupted base-pairings between the minor parental and M-/S-recombinant sequence secondary structures, whereas test 4 counted the number of disrupted base-pairings between the major parental and the M-/S-recombinant sequence secondary structures. For test 3 there was no significant evidence that fewer base-pairs present in the minor parental sequence secondary structures were disrupted in the M-recombinants than in the S-recombinants (medians of 1997 and 1990 minor parent base-pairs disrupted, respectively; p-value  = 0.328). Test 4, however, yielded marginal evidence (medians of 383 and 451.5 major parent base-pairs disrupted for the M- and S-recombinants, respectively; p-value  = 0.057) that fewer major parental sequence base-pairings were disrupted in the M-recombinants than were disrupted in the S-recombinants.

In these two tests it is entirely understandable that, relative to minor parental base-pairing disruptions, there were far fewer major parental base-pairing disruptions in both the M- and S-recombinants since by definition the M/S-recombinants are genetically far more similar to their major parents than their minor parents. It is therefore expected that, of the four tests, Test 3 would be the least likely to produce any evidence of secondary structures being less disrupted in the M-recombinants than in the S-recombinants.

It is noteworthy that these tests only indicated significant evidence of lower degrees of folding disruption in the M-recombinants than would be expected under random recombination when we considered the structure of the complete HIV-1 coding region. When we applied these and related tests to individual genome regions corresponding to known biologically functional structural elements (such as the Rev response element), they yielded no evidence that that M-recombinants had less disrupted structural elements than S-recombinants (data not shown). While it is possible that, relative to selection favouring maintenance of proper protein folding, selection favouring the maintenance of biologically functional RNA secondary structures has a much smaller influence on patterns of recombination in HIV, it is also possible that our RNA folding disruption tests were simply less powerful than the protein folding disruption tests. In this regard, the RNA folding disruption tests had four potentially important shortcomings: (1) the actual parental sequences of naturally occurring recombinants were not used in these tests and it is entirely possible that with these in hand subtle structural differences between the actual and simulated recombinant genomes would have been clearer; (2) the individual recombination events that were analysed involved single pairs of 5′ and 3′ recombination breakpoints and were probably not representative of natural recombinants which frequently have more than two detectable breakpoints; (3) the accuracy of computational secondary structure prediction is not perfect and it is likely therefore that incorrectly inferred base-pairing interactions decayed (at least slightly) the power with which disruptions of actual base-pairing interactions could be estimated; (4) the possible inclusion of non-viable viruses within the set of analysed sequences could have decreased the power of our tests because it would have violated the implicit assumption that all of the analysed HIV genomes were reasonably fit and were therefore likely free of recombination-induced RNA structure disruption. However, focusing our analyses on recombination events in CRFs (addressing shortcoming 4) and on only the most plausible biologically well characterised secondary structure elements (addressing shortcoming 3) failed to yield any stronger evidence of selection disfavouring the survival of recombinants with disrupted genomic secondary structures (data not shown).

## Conclusions

We have confirmed here that recombination events detectable in the coding regions of a number of HIV-1 proteins tend to be less disruptive of both intra-protein amino-acid – amino-acid interactions and intra-genomic nucleotide – nucleotide secondary structural interactions than would be expected if recombination were random and all recombinants were equally viable. Although this result is entirely consistent with the hypothesis that natural selection has strongly impacted the distribution of recombination events that are detectable within HIV genomes that have been sampled from the global epidemic, it does not indicate the time-scale of this selection. Specifically, while it is likely that selection over the short-term acts against newly generated recombinant genomes that have either misfolded RNA structures or express misfolded chimaeric proteins, it is similarly plausible that selection acting over the longer-term has configured the underlying structure of HIV-1M genomes so as to minimise the deleterious effects of recombination [26]. Specifically, Simon-Loriere *et. al.* have proposed that the distribution of secondary structural elements within the HIV-1M genome may maximise the chances that recombinant genomes will express properly folded chimaeric proteins by “directing” recombination breakpoints to protein domain boundaries. It remains unclear, however, how any analogous mechanism might maximise the probability of recombinant genomes having properly folded RNA secondary structures; especially since it is specifically the recombination breakpoints that occur within RNA structures that are expected to be the most disruptive of these structures. It is nevertheless possible that sequence determinants of recombination frequency besides secondary structure – such as sequence conservation [30], [31], or runs of guanosine nucleotides [32] – could also play a role in directing recombination to sites where it will have minimal impact on particular biologically functional RNA structures.
